# Hexaaqua­nickel(II) bis­{4-[(2-chlorothia­zol-5-yl)meth­oxy]benzoate} dihydrate

**DOI:** 10.1107/S1600536808007836

**Published:** 2008-03-29

**Authors:** Hong-Kun Zhang, Jin-Sheng Gao, Xiao-Hui Jiang, Guang-Feng Hou

**Affiliations:** a College of Chemistry and Chemical Engineering, China West Normal University, Nanchong 637002, People’s Republic of China; b School of Chemistry and Materials Science, Heilongjiang University, Harbin 150080, People’s Republic of China

## Abstract

In the title compound, [Ni(H_2_O)_6_](C_11_H_7_ClNO_3_S)_2_·2H_2_O, the Ni^II^ atom lies on an inversion center and is six-coordinate in an octa­hedral environment of water mol­ecules. The cation and anion are linked through O—H⋯O hydrogen bonding involving the coordinated and uncoordinated water mol­ecules into a three-dimensional network.

## Related literature

For the synthesis of 4-[(2-chloro-5-thia­zolyl)meth­oxy]benzoic acid, see: Mirci (1990 ▶). 
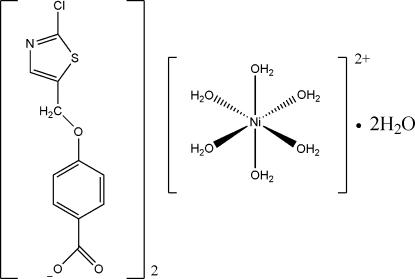


## Experimental

### 

#### Crystal data

[Ni(H_2_O)_6_](C_11_H_7_ClNO_3_S)_2_·2H_2_O
*M* *_r_* = 740.21
Triclinic, 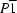

*a* = 7.1844 (4) Å
*b* = 7.2084 (4) Å
*c* = 15.5621 (8) Å
α = 78.388 (1)°
β = 81.285 (1)°
γ = 71.734 (1)°
*V* = 746.26 (7) Å^3^
*Z* = 1
Mo *K*α radiation
μ = 1.04 mm^−1^
*T* = 291 (2) K
0.20 × 0.18 × 0.08 mm

#### Data collection

Rigaku R-AXIS RAPID diffractometer
Absorption correction: multi-scan (*ABSCOR*; Higashi, 1995 ▶) *T*_min_ = 0.819, *T*_max_ = 0.921
4613 measured reflections
2862 independent reflections
2496 reflections with *I* > 2σ(*I*)
*R*_int_ = 0.012

#### Refinement

*R*[*F* ^2^ > 2σ(*F* ^2^)] = 0.030
*wR*(*F* ^2^) = 0.075
*S* = 1.06
2862 reflections
196 parameters
H-atom parameters constrained
Δρ_max_ = 0.36 e Å^−3^
Δρ_min_ = −0.22 e Å^−3^

### 

Data collection: *RAPID-AUTO* (Rigaku, 1998 ▶); cell refinement: *RAPID-AUTO*; data reduction: *CrystalStructure* (Rigaku/MSC, 2002 ▶); program(s) used to solve structure: *SHELXS97* (Sheldrick, 2008 ▶); program(s) used to refine structure: *SHELXL97* (Sheldrick, 2008 ▶); molecular graphics: *SHELXTL* (Sheldrick, 2008 ▶); software used to prepare material for publication: *SHELXL97*.

## Supplementary Material

Crystal structure: contains datablocks I. DOI: 10.1107/S1600536808007836/ng2435sup1.cif

Structure factors: contains datablocks I. DOI: 10.1107/S1600536808007836/ng2435Isup2.hkl

Additional supplementary materials:  crystallographic information; 3D view; checkCIF report

## Figures and Tables

**Table 1:** Hydrogen-bond geometry (Å, °)

*D*—H⋯*A*	*D*—H	H⋯*A*	*D*⋯*A*	*D*—H⋯*A*
O4—H8⋯O7^i^	0.85	2.05	2.903 (2)	175
O4—H9⋯O2^ii^	0.85	1.93	2.776 (2)	172
O5—H10⋯N1^iii^	0.85	2.01	2.858 (2)	173
O5—H11⋯O7^iv^	0.85	1.91	2.750 (2)	173
O6—H12⋯O1	0.85	1.94	2.781 (2)	171
O6—H13⋯O1^i^	0.85	1.90	2.747 (2)	177
O7—H14⋯O1	0.85	1.88	2.718 (2)	169

Symmetry codes: (i) 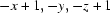; (ii) 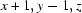; (iii) 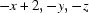; (iv) 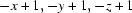.

**Fig. 1.**
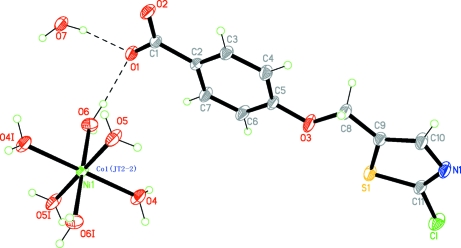
The molecular structure of (I) with the atom labeling scheme. Displacement ellipsoids are drawn at the 30% probability level. H atoms are represented as small spheres of arbitrary radii. A dashed line indicates the intermolecular O—H···O hydrogen-bonding interaction. [Symmetry code: (i) 2 - x, -y, 1 - z]

**Fig. 2.**
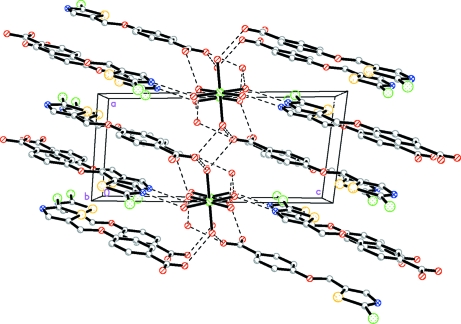
A partial packing view, showing the three-dimensional network. Dashed lines indicate the hydrogen-bonding interactions. The H atoms have been omitted for clarity.

## supplementary crystallographic information

### Comment

Simple carboxylic acids exhibit a variety of superamolecular aggregation patterns. Recenttly,our attention has been focused on 4-[(2-Chloro-5-thiazolyl)methoxy]Benzoic acid, it is a intermediate used in the synthesis of pesticide. In this paper, we reprot a new complex, (I), synthesized by the reaction of 4-[(2-chloro-5-thiazolyl)methoxy]benzoic acid and nickel(II) nitrate hexahydrate in an aqueous solution.

The asymmetric unit of (I) consists of a hexaaquanickel(II) cation, two 4-[(2-chloro-5-thiazolyl)methoxy]benzoate anions and noe uncoordinated water molecules(Fig. 1). The Ni(II) atom lies on an inversion and is coordinated by six water molecules in an octahedral environment. The anion is almost planar,the largest deviation being 0.136 (5) Å for atom Cl1.

All cations, anions and uncoordinated water molecules are linked through O—H···O hydrogen bonds,resulting in a three-dimensional supramolecular network(Fig. 2; Table 1).

### Experimental

4-[(2-Chloro-5-thiazolyl)methoxy]benzoic acid was prepared by substitute reaction of 4-hydroxybenzoic acid and 2-chloro-5-chloromethoxythiazol under basic conditions(stephen *et al.*,2000). Nickel nitrate hexahydrate(0.582 g, 2 mmol) and 4-[(2-Chloro-5-thiazolyl)methoxy]benzoic acid(0.538 g, 2 mmol) were dissolved in water(15 ml) and the pH was adjusted to 7 with 0.01 mol/*L* sodium hydroxide. Jade-green crystals separated from filtered after several days.

### Refinement

H atoms bound to C atoms were placed in calculated positions and treated as riding on their parent atoms, with C—H = 0.93 Å (aromatic C), C—H = 0.97 Å (methylene C), and with *U*_iso_(H) = 1.2Ueq(C). Water H atoms were initially located in a difference Fourier map but they were treated as riding on their parent atoms with O—H=0.85 Å, *U*_iso_(H) = 1.5Ueq(O).

### Crystal data

**Table:**

[Ni(H_2_O)_6_](C_11_H_7_ClNO_3_S)_2_·2H_2_O	*Z* = 1
*M**_r_* = 740.21	*F*_000_ = 382
Triclinic, *P*1	*D*_x_ = 1.647 Mg m^−^^3^
Hall symbol: -P 1	Mo *K*α radiation λ = 0.71073 Å
*a* = 7.1844 (4) Å	Cell parameters from 4613 reflections
*b* = 7.2084 (4) Å	θ = 2.7–28.2º
*c* = 15.5621 (8) Å	µ = 1.04 mm^−^^1^
α = 78.388 (1)º	*T* = 291 (2) K
β = 81.285 (1)º	Block, green
γ = 71.734 (1)º	0.20 × 0.18 × 0.08 mm
*V* = 746.26 (7) Å^3^	

### Data collection

**Table:**

Rigaku R-AXIS RAPID diffractometer	2862 independent reflections
Radiation source: fine-focus sealed tube	2496 reflections with *I* > 2σ(*I*)
Monochromator: graphite	*R*_int_ = 0.012
*T* = 291(2) K	θ_max_ = 26.0º
ω scan	θ_min_ = 2.7º
Absorption correction: multi-scan(ABSCOR; Higashi, 1995)	*h* = −8→8
*T*_min_ = 0.819, *T*_max_ = 0.922	*k* = −4→8
4613 measured reflections	*l* = −19→19

### Refinement

**Table:**

Refinement on *F*^2^	Secondary atom site location: difference Fourier map
Least-squares matrix: full	Hydrogen site location: inferred from neighbouring sites
*R*[*F*^2^ > 2σ(*F*^2^)] = 0.030	H-atom parameters constrained
*wR*(*F*^2^) = 0.076	*w* = 1/[σ^2^(*F*_o_^2^) + (0.0324*P*)^2^ + 0.4113*P*] where *P* = (*F*_o_^2^ + 2*F*_c_^2^)/3
*S* = 1.06	(Δ/σ)_max_ < 0.001
2862 reflections	Δρ_max_ = 0.36 e Å^−^^3^
196 parameters	Δρ_min_ = −0.22 e Å^−^^3^
Primary atom site location: structure-invariant direct methods	Extinction correction: none

### Special details

**Table:**

Geometry. All e.s.d.'s (except the e.s.d. in the dihedral angle between two l.s. planes) are estimated using the full covariance matrix. The cell e.s.d.'s are taken into account individually in the estimation of e.s.d.'s in distances, angles and torsion angles; correlations between e.s.d.'s in cell parameters are only used when they are defined by crystal symmetry. An approximate (isotropic) treatment of cell e.s.d.'s is used for estimating e.s.d.'s involving l.s. planes.
Refinement. Refinement of *F*^2^ against ALL reflections. The weighted *R*-factor *wR* and goodness of fit *S* are based on *F*^2^, conventional *R*-factors *R* are based on *F*, with *F* set to zero for negative *F*^2^. The threshold expression of *F*^2^ > σ(*F*^2^) is used only for calculating *R*-factors(gt) *etc*. and is not relevant to the choice of reflections for refinement. *R*-factors based on *F*^2^ are statistically about twice as large as those based on *F*, and *R*- factors based on ALL data will be even larger.

### Fractional atomic coordinates and isotropic or equivalent isotropic displacement parameters (Å^2^)

**Table:**

	*x*	*y*	*z*	*U*_iso_*/*U*_eq_	
C1	0.4407 (3)	0.4075 (3)	0.34973 (14)	0.0299 (5)	
C2	0.5199 (3)	0.3065 (3)	0.27064 (14)	0.0290 (4)	
C3	0.5491 (3)	0.4165 (3)	0.18875 (14)	0.0341 (5)	
H1	0.5231	0.5528	0.1836	0.041*	
C4	0.6166 (3)	0.3275 (3)	0.11385 (15)	0.0377 (5)	
H2	0.6367	0.4031	0.0593	0.045*	
C5	0.6532 (3)	0.1257 (3)	0.12194 (14)	0.0357 (5)	
C6	0.6261 (4)	0.0128 (3)	0.20322 (15)	0.0454 (6)	
H3	0.6515	−0.1234	0.2081	0.055*	
C7	0.5612 (4)	0.1027 (3)	0.27714 (15)	0.0414 (6)	
H4	0.5450	0.0260	0.3318	0.050*	
C8	0.7285 (4)	0.1271 (3)	−0.03345 (14)	0.0404 (5)	
H6	0.8182	0.2060	−0.0388	0.048*	
H5	0.5999	0.2151	−0.0474	0.048*	
C9	0.8018 (3)	−0.0208 (3)	−0.09460 (14)	0.0336 (5)	
C10	0.8419 (4)	0.0072 (4)	−0.18299 (15)	0.0444 (6)	
H7	0.8252	0.1330	−0.2162	0.053*	
C11	0.9178 (3)	−0.3136 (3)	−0.16161 (15)	0.0369 (5)	
Cl1	0.99611 (11)	−0.54983 (9)	−0.18443 (5)	0.05607 (19)	
N1	0.9093 (3)	−0.1615 (3)	−0.22133 (13)	0.0431 (5)	
Ni1	1.0000	0.0000	0.5000	0.02618 (11)	
O1	0.4168 (2)	0.2996 (2)	0.42325 (10)	0.0360 (4)	
O2	0.4022 (2)	0.5919 (2)	0.33923 (10)	0.0420 (4)	
O3	0.7165 (3)	0.0213 (2)	0.05258 (10)	0.0521 (5)	
O4	1.0411 (2)	−0.1930 (2)	0.41173 (11)	0.0435 (4)	
H8	0.9563	−0.2515	0.4097	0.065*	
H9	1.1530	−0.2663	0.3946	0.065*	
O5	0.9559 (3)	0.2269 (2)	0.39824 (11)	0.0519 (5)	
H10	1.0058	0.2093	0.3463	0.078*	
H11	0.8994	0.3483	0.4014	0.078*	
O6	0.7076 (2)	0.0327 (2)	0.52300 (11)	0.0403 (4)	
H12	0.6286	0.1176	0.4888	0.061*	
H13	0.6671	−0.0686	0.5411	0.061*	
O7	0.2566 (2)	0.3897 (2)	0.58489 (11)	0.0418 (4)	
H15	0.3537	0.3765	0.6131	0.063*	
H14	0.3016	0.3778	0.5319	0.063*	
S1	0.84843 (10)	−0.26984 (9)	−0.05532 (4)	0.04168 (16)	

### Atomic displacement parameters (Å^2^)

**Table:**

	*U*^11^	*U*^22^	*U*^33^	*U*^12^	*U*^13^	*U*^23^
C1	0.0288 (10)	0.0311 (12)	0.0304 (11)	−0.0066 (9)	−0.0005 (8)	−0.0112 (9)
C2	0.0305 (10)	0.0267 (11)	0.0279 (11)	−0.0039 (8)	−0.0003 (8)	−0.0090 (9)
C3	0.0447 (12)	0.0248 (11)	0.0319 (12)	−0.0084 (9)	0.0001 (9)	−0.0082 (9)
C4	0.0515 (13)	0.0326 (12)	0.0258 (11)	−0.0108 (10)	0.0030 (10)	−0.0049 (9)
C5	0.0450 (12)	0.0313 (12)	0.0270 (11)	−0.0040 (10)	0.0032 (9)	−0.0126 (9)
C6	0.0708 (17)	0.0232 (12)	0.0336 (13)	−0.0049 (11)	0.0067 (12)	−0.0080 (10)
C7	0.0600 (15)	0.0301 (12)	0.0268 (11)	−0.0074 (11)	0.0080 (10)	−0.0065 (10)
C8	0.0577 (14)	0.0332 (13)	0.0272 (12)	−0.0087 (11)	0.0025 (10)	−0.0104 (10)
C9	0.0417 (12)	0.0282 (11)	0.0291 (11)	−0.0076 (9)	0.0003 (9)	−0.0075 (9)
C10	0.0702 (16)	0.0297 (12)	0.0284 (12)	−0.0098 (11)	0.0028 (11)	−0.0071 (10)
C11	0.0434 (12)	0.0328 (12)	0.0327 (12)	−0.0067 (10)	0.0022 (10)	−0.0126 (10)
Cl1	0.0756 (5)	0.0341 (3)	0.0554 (4)	−0.0099 (3)	0.0077 (3)	−0.0205 (3)
N1	0.0622 (13)	0.0352 (11)	0.0283 (10)	−0.0085 (9)	0.0044 (9)	−0.0130 (9)
Ni1	0.0301 (2)	0.0224 (2)	0.0240 (2)	−0.00492 (15)	0.00206 (14)	−0.00691 (15)
O1	0.0464 (9)	0.0331 (8)	0.0274 (8)	−0.0104 (7)	0.0037 (7)	−0.0098 (7)
O2	0.0575 (10)	0.0278 (8)	0.0362 (9)	−0.0050 (7)	0.0040 (7)	−0.0130 (7)
O3	0.0902 (14)	0.0318 (9)	0.0255 (8)	−0.0070 (9)	0.0071 (8)	−0.0114 (7)
O4	0.0432 (9)	0.0421 (10)	0.0493 (10)	−0.0121 (7)	0.0064 (7)	−0.0255 (8)
O5	0.0836 (13)	0.0268 (9)	0.0277 (8)	0.0023 (8)	0.0087 (8)	−0.0048 (7)
O6	0.0340 (8)	0.0358 (9)	0.0482 (10)	−0.0101 (7)	0.0004 (7)	−0.0035 (7)
O7	0.0494 (9)	0.0378 (9)	0.0371 (9)	−0.0085 (7)	0.0032 (7)	−0.0159 (7)
S1	0.0599 (4)	0.0318 (3)	0.0279 (3)	−0.0089 (3)	0.0050 (3)	−0.0064 (2)

### Geometric parameters (Å, °)

**Table:**

C1—O2	1.251 (3)	C10—N1	1.380 (3)
C1—O1	1.269 (3)	C10—H7	0.9300
C1—C2	1.503 (3)	C11—N1	1.279 (3)
C2—C3	1.383 (3)	C11—Cl1	1.710 (2)
C2—C7	1.390 (3)	C11—S1	1.716 (2)
C3—C4	1.393 (3)	Ni1—O5	2.0167 (16)
C3—H1	0.9300	Ni1—O5^i^	2.0167 (16)
C4—C5	1.378 (3)	Ni1—O6^i^	2.0230 (15)
C4—H2	0.9300	Ni1—O6	2.0230 (15)
C5—O3	1.379 (3)	Ni1—O4^i^	2.0732 (15)
C5—C6	1.382 (3)	Ni1—O4	2.0732 (15)
C6—C7	1.381 (3)	O4—H8	0.8499
C6—H3	0.9300	O4—H9	0.8500
C7—H4	0.9300	O5—H10	0.8500
C8—O3	1.406 (3)	O5—H11	0.8500
C8—C9	1.492 (3)	O6—H12	0.8499
C8—H6	0.9700	O6—H13	0.8500
C8—H5	0.9700	O7—H15	0.8499
C9—C10	1.349 (3)	O7—H14	0.8501
C9—S1	1.718 (2)		
			
O2—C1—O1	124.10 (19)	N1—C10—H7	121.9
O2—C1—C2	118.33 (19)	N1—C11—Cl1	122.70 (17)
O1—C1—C2	117.58 (19)	N1—C11—S1	116.45 (17)
C3—C2—C7	118.43 (19)	Cl1—C11—S1	120.85 (14)
C3—C2—C1	120.19 (19)	C11—N1—C10	109.39 (19)
C7—C2—C1	121.36 (19)	O5—Ni1—O5^i^	180.000 (1)
C2—C3—C4	121.4 (2)	O5—Ni1—O6^i^	88.79 (7)
C2—C3—H1	119.3	O5^i^—Ni1—O6^i^	91.21 (7)
C4—C3—H1	119.3	O5—Ni1—O6	91.21 (7)
C5—C4—C3	118.9 (2)	O5^i^—Ni1—O6	88.79 (7)
C5—C4—H2	120.5	O6^i^—Ni1—O6	180.00 (9)
C3—C4—H2	120.5	O5—Ni1—O4^i^	91.14 (7)
C4—C5—O3	124.3 (2)	O5^i^—Ni1—O4^i^	88.86 (7)
C4—C5—C6	120.6 (2)	O6^i^—Ni1—O4^i^	92.92 (6)
O3—C5—C6	115.1 (2)	O6—Ni1—O4^i^	87.08 (6)
C7—C6—C5	119.9 (2)	O5—Ni1—O4	88.86 (7)
C7—C6—H3	120.0	O5^i^—Ni1—O4	91.14 (7)
C5—C6—H3	120.0	O6^i^—Ni1—O4	87.08 (6)
C6—C7—C2	120.7 (2)	O6—Ni1—O4	92.92 (6)
C6—C7—H4	119.6	O4^i^—Ni1—O4	180.0
C2—C7—H4	119.6	C5—O3—C8	118.64 (18)
O3—C8—C9	107.34 (18)	Ni1—O4—H8	121.8
O3—C8—H6	110.2	Ni1—O4—H9	123.8
C9—C8—H6	110.2	H8—O4—H9	107.5
O3—C8—H5	110.2	Ni1—O5—H10	121.0
C9—C8—H5	110.2	Ni1—O5—H11	126.5
H6—C8—H5	108.5	H10—O5—H11	112.2
C10—C9—C8	129.8 (2)	Ni1—O6—H12	120.0
C10—C9—S1	109.38 (17)	Ni1—O6—H13	119.8
C8—C9—S1	120.84 (16)	H12—O6—H13	109.8
C9—C10—N1	116.1 (2)	H15—O7—H14	107.2
C9—C10—H7	121.9	C11—S1—C9	88.63 (11)

Symmetry codes: (i) −*x*+2, −*y*, −*z*+1.

### Hydrogen-bond geometry (Å, °)

**Table:**

*D*—H···*A*	*D*—H	H···*A*	*D*···*A*	*D*—H···*A*
O4—H8···O7^ii^	0.85	2.05	2.903 (2)	175
O4—H9···O2^iii^	0.85	1.93	2.776 (2)	172
O5—H10···N1^iv^	0.85	2.01	2.858 (2)	173
O5—H11···O7^v^	0.85	1.91	2.750 (2)	173
O6—H12···O1	0.85	1.94	2.781 (2)	171
O6—H13···O1^ii^	0.85	1.90	2.747 (2)	177
O7—H14···O1	0.85	1.88	2.718 (2)	169

Symmetry codes: (ii) −*x*+1, −*y*, −*z*+1; (iii) *x*+1, *y*−1, *z*; (iv) −*x*+2, −*y*, −*z*; (v) −*x*+1, −*y*+1, −*z*+1.
